# DSLHash: Lightweight Hash Function Based on SVSC 4D Chaotic System and Dynamic Substitution Layers

**DOI:** 10.3390/e28090974

**Published:** 2026-09-02

**Authors:** Jun Zhang, Xiangping Li, Yu Zeng, Xingbin Wang, Yunzheng Yang, Yun Wang, Chaozhong Wu

**Affiliations:** 1 Hubei Key Laboratory of Vehicle-Infrastructure Collaboration and Traffic Control, Hubei University of Arts and Science, Xiangyang 441053, China; lixiangping@hbuas.edu.cn (X.L.); 15025433425@163.com (Y.Y.); wangyun@hbuas.edu.cn (Y.W.); wucz@whut.edu.cn (C.W.); 2 Research Institute of Smart Transportation, Hubei University of Arts and Science, Xiangyang 441053, China; 3 School of Artificial Intelligence and Automation, Huazhong University of Science and Technology, Wuhan 430074, China; zedoth777@gmail.com; 4 School of Cybersecurity, Tianjin University, Tianjin 300350, China; wangxingbin@tju.edu.cn

**Keywords:** lightweight hash function, sponge construction, 4D chaotic map, dynamic substitution layer

## Abstract

Data integrity remains a critical requirement for secure communication in IoT environments. To address this challenge for resource-constrained IoT devices, lightweight cryptographic hash functions have been widely studied. However, static substitution layers in conventional hash functions might be vulnerable to advanced cryptanalysis. Dynamic substitution layers are constructed by exploring several methods such as key dependency, chaotic maps, DNA computations, and elliptic curves. Despite these efforts, existing dynamic substitution layers are not suitable for resource-efficient implementation. To achieve both enhanced security and practicality, this paper proposes a lightweight hash function based on a structure-varying self-coupled (SVSC) chaotic system and a dynamic substitution mechanism. A SVSC 4D chaotic map is first constructed using lightweight arithmetic and logical operations, including modular addition, bitwise shifts, and bitwise negation, thereby avoiding multiplication operations and enabling efficient hardware implementation. The chaotic map is then integrated with a generalized Feistel structure to construct a dynamic substitution layer, where the transformation behavior dynamically changes according to the internal chaotic state. This mechanism enhances nonlinear transformation diversity and contributes to improved diffusion characteristics. Finally, the dynamic substitution layer is incorporated into a sponge-based hash framework supporting flexible digest lengths. The results demonstrate that the proposed SVSC 4D chaotic system exhibits long cycle lengths in a 32-bit finite-precision implementation, mitigating the degradation caused by finite-precision effects and improving its suitability for practical cryptographic applications. Experimental evaluations demonstrate that the proposed methodology achieves a balanced tradeoff among security performance, hardware efficiency, and implementation complexity. Based on a 256-bit sponge state with a 192-bit capacity, DSLHash provides approximately 96-bit collision resistance, which is selected to balance security requirements and hardware resource consumption in lightweight applications.

## 1. Introduction

The Internet of Things (IoT) represents a transformative technology that enables seamless connectivity among diverse smart devices and facilitates intelligent data exchange through the internet. According to authoritative projections by Statista and Gartner [1], the global IoT device count will surpass 32.1 billion by 2030, while the market is anticipated to reach $991 billion by 2028 [2]. This exponential growth in device interconnectivity and data volume introduces profound security challenges, with data integrity emerging as a foundational requirement for secure IoT communications [3,4]. Cryptographic hash functions serve as fundamental security primitives that convert variable-length inputs into fixed-size deterministic hash values. These primitives play a pivotal role in ensuring message integrity, authenticating entities, verifying passwords, and securing blockchain systems [4,5,6].

Traditional cryptographic hash functions like SHA-3, designed for general-purpose computing environments, may not be suitable for resource-constrained IoT devices. These devices typically exhibit extreme limitations in processing power, memory footprint, and energy budget [7,8]. For instance, remote weather/hydrologic stations require ultra-low power consumption and compact hardware implementations [9], and health sensor networks depend on implantable/wearable medical devices with strict energy and area constraints to operate continuously in diverse environments [3]. For this reason, the research community has prioritized the development of lightweight cryptographic hash functions (LWHFs) that balance security with resource efficiency.

Within Substitution–Permutation Network (SPN)-based LWHFs [10,11,12], the substitution layer serves as the primary nonlinear component responsible for introducing confusion and facilitating diffusion. The effectiveness of information propagation throughout the cryptographic state is therefore closely related to the design quality of the substitution mechanism. However, conventional static substitution layers or fixed S-boxes suffer from several inherent limitations, including fixed points, inverse fixed points, and short-cycle structures [13,14]. More importantly, the repeated use of an identical substitution structure across all rounds may restrict the diversity of state transformations, leading to predictable patterns and potentially exploitable cryptanalytic characteristics [13].

To address these limitations, researchers have explored dynamic substitution layer design paradigms, including randomness [15], key-dependent permutations [16,17], elliptic curve-based nonlinearities [18], and chaotic systems [19,20,21]. By dynamically altering the substitution structure in each permutation round, these approaches inherently mitigate vulnerabilities associated with static S-boxes (e.g., fixed points, limited cycle periods) and enhance resistance to differential cryptanalysis [22]. Despite these security improvements, existing dynamic substitution implementations remain unsuitable for lightweight hash functions. Notably, chaos-based designs often rely on floating-point arithmetic [23,24,25] and multiplier operations, which introduce significant hardware resource overhead and computational complexity that are incompatible with IoT constraints [26].

The main contributions of this work are summarized as follows:

- A multiplier-free SVSC 4D chaotic map is proposed by dynamically coupling four 1D chaotic maps with a 32-bit finite-precision integer implementation. The results demonstrate long cycle lengths and strong robustness against finite-precision effects, highlighting its potential for practical cryptographic applications.
- A dynamic substitution layer is developed by integrating the proposed SVSC chaotic map with a Type-II generalized Feistel structure (GFS) [27]. Based on this design, a lightweight hash function, termed DSLHash, is constructed within the sponge framework to improve information diffusion and confusion.
- Security and hardware evaluations indicate that DSLHash provides favorable statistical randomness, efficient diffusion and confusion characteristics, and a compact FPGA implementation, offering a practical security–efficiency trade-off for resource-constrained IoT applications.

The remaining sections of this paper are organized as follows: Section 2 provides the background and preliminary information for the lightweight hash function, and Section 3 presents the construction process of the SVSC 4D chaotic map. The proposed lightweight hash function is then detailed in Section 4. Section 5 and Section 6 provide the security analysis and hardware implementation for the proposed hash function. Finally, this paper is concluded in Section 7.

## 2. Background and Preliminaries

This section provides a comprehensive overview of lightweight hash functions, the dynamic substitution layer and the sponge construction, analyzing their structural design principles and security characteristics.

### 2.1. Lightweight Hash Function

In 2018, NIST launched the Lightweight Cryptography (LWC) standardization process to address the growing demand for cryptographic algorithms that balance security and resource efficiency. This initiative led to the announcement of ten finalists in March 2021 [28]. These include prominent candidates such as: ASCON, Elephant, GIFT-COFB, Grain128-AEAD, ISAP, Photon-Beetle, Romulus, Sparkle, TinyJambu, and Xoodyak [28]. Among the finalists, Ascon emerged as the NIST LWC winner [10], demonstrating exceptional performance in terms of resource efficiency and security margins. Meanwhile, Photon-Beetle and SPONGENT achieved international recognition through ISO/IEC certification [11,12], solidifying their roles as foundational primitives for IoT security protocols.

The sponge construction and its variants have become foundational frameworks for developing lightweight hash functions due to their proven security guarantees and computational efficiency [10,11,12]. These constructions typically utilize permutation functions to process their internal state through iterative SPN-based round transformations. Examples of such hash functions include Ascon-Hash, Xoodyak, Photon-Beetle-Hash, SPARKLE, SPONGENT, and Gimli-Hash [28]. A critical component of these designs is the substitution layer, often realized as an affine-equivalent S-box, which serves as the sole nonlinear element in the transformation process. Photon-Beetle-Hash employs a 256-bit permutation to process a state composed of 64 × 4-bit cells, applying a 4-bit S-box to each cell individually [29]. The Ascon family’s substitution layer features 64 parallel instances of a 5-bit S-box, derived from Keccak’s χ mapping [30]. By applying the S-box to each bit slice of its five internal registers, the design achieves hardware-friendly parallelism, reducing latency and power consumption. SPARKLE implements its substitution layer using the ARX-box construct [31], realized through the Alzette iterated block cipher. Alzette’s four-round structure employs variable rotation amounts in each round, creating nonlinear transformations with strong differential resistance.

### 2.2. Dynamic S-Box and Substitution Layer

The substitution layer (S-box), as the sole nonlinear component in cryptographic hash functions, plays a pivotal role in realizing both diffusion and confusion properties—critical for achieving algorithmic security. While static S-boxes employed in standards like AES and SM4 exhibit high nonlinearity (112 bits for AES), there exist some weaknesses in existing S-boxes, such as fixed points, reverse fixed points, and short iteration period rings [13,14]. A fundamental limitation of static substitution layers is their fixed structural repetition across rounds. This repetition introduces predictable patterns susceptible to cryptanalysis, as adversaries can exploit invariant transformations to derive key insights [13,14]. In contrast, dynamic substitution layers dynamically alter their structure in each permutation round, thereby eliminating fixed points and periodicity by continuously reshaping the nonlinear mapping, and better resisting against various cryptanalysis attacks [32].

Researchers have investigated multiple innovative dynamic S-box design techniques to address the limitations of static implementations. Key approaches include leveraging randomness and key dependency mechanisms to enhance security properties [13]. For instance, Adi Reddy et al. used a random number generator for sub-keys in the key expansion module of their algorithm [15]. The work showed that the proposed S-boxes are free from linear and differential cryptanalysis attacks. Krishnamurthy et al. employed S-box rotation and used it as an additional component in the traditional AES algorithm to design a dynamic S-box [16]. The process consists of three steps in which the S-boxes are rotated based on fixed, partial, and whole key values to increase their security. Arrag et al. extended and modified the AES key expansion schedule and used an S-box lookup table to construct a dynamic S-box with good nonlinearity [17]. Hussain et al. proposed a dynamic S-box algorithm based on a special supersingular elliptic curve (EC) [18]. They introduced novel models within a special supersingular EC framework and generated the S-box as a complementary element.

The inherent properties of chaotic systems —including initial state sensitivity, unpredictability, and parameter controllability —align seamlessly with cryptographic requirements for confusion and diffusion [19,20,22]. This synergy has driven extensive research on chaos-based dynamic S-box designs, which offer unique advantages over traditional static implementations. Açikkapi et al. conducted a comprehensive side-channel analysis comparing S-box implementations based on chaotic maps versus traditional S-boxes in the AES algorithm. Their evaluation revealed that chaotic-map-derived S-boxes exhibit significantly enhanced resistance to side-channel attacks [22]. Duong et al. proposed an effective method to create a 4 × 4 S-box based on an enhanced tent map [19]. Gaabouri et al. presented a novel method for S-box pattern generation using a chaos-enhanced logistic map [20]. Their design emphasizes resource efficiency by constructing a 5-bit S-box optimized for lightweight cryptosystems. Ma et al. proposed a dynamic S-box construction method leveraging the two-dimensional hyperchaotic effect within a coupled map lattice system [21].

Instead of relying on chaotic maps for dynamic S-box design, several attempts were made to design dynamic substitution layers based on chaotic sponge construction. Teh et al. presented an unkeyed hash function utilizing a spatiotemporal coupling of a 4D tent map within a sponge framework [23]. Their implementation employs fixed-point arithmetic to avoid the computational overhead associated with floating-point operations, thereby optimizing performance for applications. Alawida et al. used DNA sequences to design state transition rules to enhance the properties of digital chaotic maps and proposed a hash function based on chaotic sponge construction [24]. Abdoun et al. implemented two secure keyed hash functions based on sponge construction and the chaotic neural network [25]. While existing dynamic substitution layers based on chaotic maps have enhanced the security and efficiency of hash functions, they remain unsuitable for resource-constrained systems due to inherent computational complexity. This paper proposes a novel dynamic substitution layer constructed from a SVSC 4D chaotic map, specifically designed to address the limitations of lightweight hash functions in IoT environments.

### 2.3. Sponge Construction

The sponge structure is a fundamental cryptographic primitive that employs fixed-length transformation processes to map variable-length binary input sequences $Z_{2}^{*}$ to fixed-length binary output sequences $Z_{2}^{n}$ [33,34]. This design serves as the foundational building block for critical cryptographic primitives, including hash functions and authenticated encryption schemes. The sponge structure mainly consists of three parts. One part is the sponge state *S* with *s* bits. *S* is divided into two parts: the bit rate $b_{r}$ and the capacity *c*. Among them, the number of bits *r* in $b_{r}$ determines the speed of processing the input information. While *c* has nothing to do with the number of bits of the input and output, it determines the security boundary of the entire sponge function. The second part is the function Γ that transforms the sponge state: $\Gamma :{\left\{0,1\right\}}^{s}\to {\left\{0,1\right\}}^{s}$. Γ is a pseudo-random permutation algorithm that transforms *S* and realizes the nonlinear mixing of the $b_{r}$ bits and the *c* bits. The last part is the method of padding the input message to an integer multiple of *s*.

**Figure 1:**
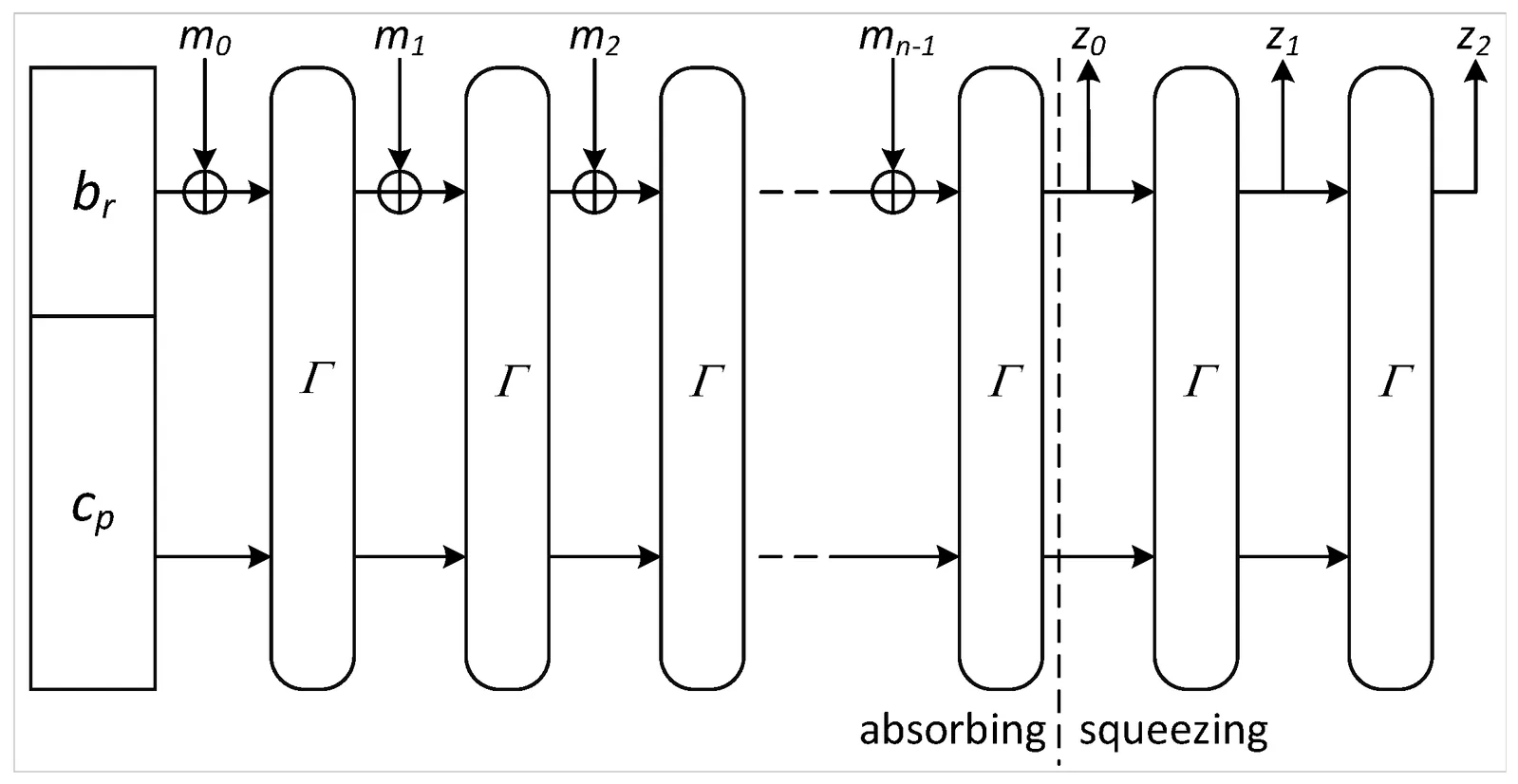
The sponge construction used in this paper.

As shown in Figure 1, the sponge structure is divided into two phases: the absorption phase and the squeezing phase. During the absorption phase, the data $m_{i}$ from the input message is XORed with $b_{r}$. Then the entire sponge state *S* is processed by the permutation function Γ to mix $b_{r}$ and the *c*. This process is repeated continuously until all the input data has been processed. In the squeezing phase, the entire sponge state is also transformed by the permutation function Γ, and a certain number of bits are extracted as the output result. This process is repeated continuously as well until the output of the predetermined length is obtained.

## 3. SVSC Chaotic Map

As detailed in Section 2.2, chaotic maps have emerged as critical components in cryptographic primitives due to their inherent unpredictability and sensitivity to initial conditions. Their security levels are strongly dependent on chaotic performances. As one-dimensional (1D) chaotic maps exhibit many drawbacks, such as low complexity and vulnerability to dynamical degradation, researchers focus on studying high-dimensional (HD) chaotic maps, which have more complex structures and better chaotic performances [35]. However, their practical implementation in LWC faces critical challenges. HD chaotic maps typically require floating-point arithmetic and multiplication operations, which incur significant hardware resource and energy consumption in resource-constrained environments like IoT.

To design a lightweight hash function with ease of implementation in mind, we propose a SVSC 4D chaotic map constructed using the methodology proposed by Hu et al. [35,36]. According to their method, when $f:S_{1}\to S_{1}$ is a self-map with $L_{i}$ denoting the infimum of its derivatives magnitudes, a composite self-map $F=f_{0}\times f_{1}\times \cdot \cdot \cdot \times f_{n-1}$ is defined, and the minimum infimum is $L=min\{L_{0},L_{1},\ldots L_{n-1}\}$. Let T(x)=Ax(mod1) be a self-map, *A* is an integer matrix with entries in $Z_{2}^{n\times n}$ and μ is the minimum singular value of *A*. F∘T is a dispersal map when $L\mu >2\sqrt{n}$. Based on this general method, a SVSC 4D chaotic map can be constructed by the following steps.

Given the functions $f_{i}$ (i=0,1,2,3) defined as

(1) $$f_{i}=\left\{\begin{array}{cc} \left(73+2^{-28}\right)x\left(mod\;1\right) & :\;0\leq x<i/5\\ 1-(\left(73+2^{-28}\right)x\left(mod\;1\right)) & :\;i/5\leq x<1 \end{array}\right.\;\;,\;x\in \left[0,1\right),$$

and the integer matrix *A* defined as

(2) $$A=\left[\begin{array}{cccc} 1 & 1 & 0 & 0\\ 0 & 1 & 1 & 0\\ 0 & 0 & 1 & 1\\ 0 & 1 & 1 & 1 \end{array}\right],$$

and their composition $F=f_{0}\times f_{1}\times f_{2}\times f_{3}$, we have $L=73+2^{-28}$ and μ≈0.3609. Since $L\mu \approx 26.35>2\sqrt{4}=4$, a 4D chaotic map can be constructed by F∘T. Furthermore, for any rational number x∈[0,1), it can be expressed in finite binary form: $x={\left(0.x_{1}x_{2}\ldots x_{n}\right)}_{2}=\sum_{i=1}^{n}\frac{x_{i}}{2^{i}}$ ($x_{i}\in \left\{0,1\right\}$). When n=32, the rational number is represented as a 32-bit binary $x_{32}=\left(0.x_{1}x_{2}\ldots x_{32}\right)$. As decimal is not suitable for hardware implementation, we apply map $\sigma :x_{32}^{\prime }\to 2^{32}x_{32}$ to $x_{32}$, which scales $x_{32}$ to the range [0, 2^32^) [37,38]. Then, Equation (1) is transformed to

(3) $$f_{i}^{\prime }\left(x\right)=\left\{\begin{array}{cc} \left(x\ll 6)⊞(x\ll 3)⊞x⊞(x\gg 28\right) & :\;0\leq x\leq \left\lfloor 2^{32}*i/5\right\rfloor \\ \bar{\left(x\ll 6)⊞(x\ll 3)⊞x⊞(x\gg 28\right)}⊞1 & :\;\left\lfloor 2^{32}*i/5\right\rfloor +1\leq x<2^{32} \end{array}\right..$$

and the resulting 4D chaotic map (called as Type-0) can be represented as

(4) $$X_{i+1}=F\left(\left(AX_{i}\right)\left(mod\;2^{32}\right)\right).$$

In this way, the proposed lightweight chaotic map can be efficiently implemented using only modular addition, bitwise shifts, and bitwise inversion as outlined in Algorithm 1 (ChaoticMap1D). This design eliminates resource-intensive components such as multipliers, making it highly amenable to hardware realization. It should be noted that other methods can also be used to develop the 4D chaotic system [39].

To enable dynamic structural adaptation of the SVSC 4D chaotic map, the most straightforward approach is to determine the coupling structure by the current chaotic state [23,40]. Since the chaotic state exhibits random and uniform distribution, we select specific bits from the state vector $f_{i}$ as control parameters for the coupling structure, as implemented in Algorithm 1. The first strategy for configuring the coupling structure involves applying random permutations to the chaotic state variables $X_{i}$ (i = 0,1,2,3). This operation is implemented through dot multiplication of the state variables with the permutation matrix $A_{1k}$ defined as

(5) $$A_{1k}={\left[\begin{array}{cccc} 0 & 1 & 0 & 0\\ 0 & 0 & 1 & 0\\ 0 & 0 & 0 & 1\\ 1 & 0 & 0 & 0 \end{array}\right]}^{\left(2k[7]+k[0]\right)},$$

and the SVSC 4D chaotic map (Type-1) can be represented as

(6) $$X_{i+1}=F\left(A\left(A_{1k}X_{i}\right)\left(mod\;2^{32}\right)\right).$$

**Algorithm 1:** Structure-varying high-dimensional chaotic map
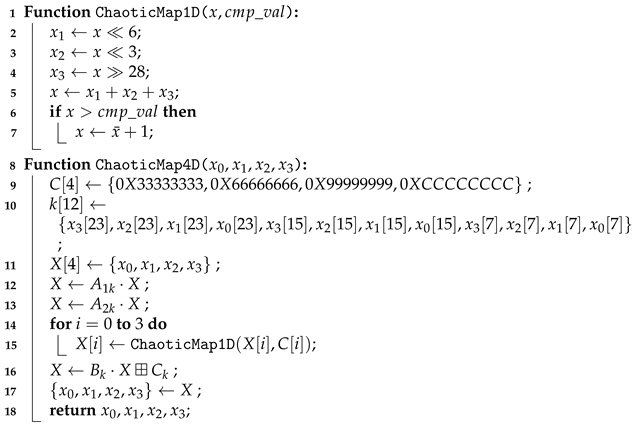


Then, we introduce a mechanism to modify the integer matrix *A* using the control parameters. The new couple matrix $A_{2k}$ is expressed as

(7) $$A_{2k}=\left[\begin{array}{cccc} {\left(-1\right)}^{k[7]} & {\left(-1\right)}^{k[0]} & 0 & 0\\ 0 & {\left(-1\right)}^{k[6]} & {\left(-1\right)}^{k[1]} & 0\\ 0 & 0 & {\left(-1\right)}^{k[5]} & {\left(-1\right)}^{k[2]}\\ 0 & {\left(-1\right)}^{k[3]} & {\left(-1\right)}^{k[3]} & {\left(-1\right)}^{k[4]} \end{array}\right],$$

and the corresponding SVSC 4D chaotic map (Type-2) can be represented as

(8) $$X_{i+1}=F\left(\left(A_{2k}A_{1k}X_{i}\right)\left(mod\;2^{32}\right)\right).$$

The dynamic coupling matrix $A_{2k}A_{1k}$ is determined by 8 control variables, resulting in 256 possible coupling matrices. An exhaustive enumeration was implemented in Python 3.11 to compute the singular values of all possible coupling matrices. The smallest singular-value lower bound among all coupling matrices was obtained as μ≈0.2451 (Lμ≈17.89>4), indicating that all possible coupling configurations maintain a non-degenerate coupling strength.

Finally, to augment the nonlinear complexity of the SVSC 4D chaotic map, we introduce a bitwise inversion mechanism for each sub-chaotic map $f_{i}$, controlled by a state-derived control parameter $k_{i}$. When the control parameter evaluates to true (i.e., $k_{i}=1$), the state of $f_{i}$ undergoes bitwise inversion, transforming $f_{i}$ into $\bar{f_{i}}=1-f_{i}$. This process is achieved through dot multiplying with matrix $B_{k}$ and modular adding with matrix $C_{k}$, which are defined as

(9) $$B_{k}=\left[\begin{array}{cccc} {\left(-1\right)}^{k[8]} & 0 & 0 & 0\\ 0 & {\left(-1\right)}^{k[9]} & 0 & 0\\ 0 & 0 & {\left(-1\right)}^{k[10]} & 0\\ 0 & 0 & 0 & {\left(-1\right)}^{k[11]} \end{array}\right],C_{k}=\left[\begin{array}{c} -k[8]\\ -k[9]\\ -k[10]\\ -k[11] \end{array}\right].$$

Then the SVSC 4D chaotic map (Type-3) can be represented as

(10) $$X_{i+1}=B_{k}F\left(A_{2k}\left(A_{1k}X_{i}\right)\left(mod\;2^{32}\right)\right)⊞C_{k},$$

and the details are illustrated in Algorithm 1. Since $B_{k}$ is a diagonal sign matrix with entries of ±1, it is an orthogonal matrix satisfying $B_{k}^{T}B_{k}=I$, which does not change the singular values of the coupling matrix. Therefore, the dispersal property of the dynamic coupling matrix is preserved after applying the sign transformation.

**Figure 2:**
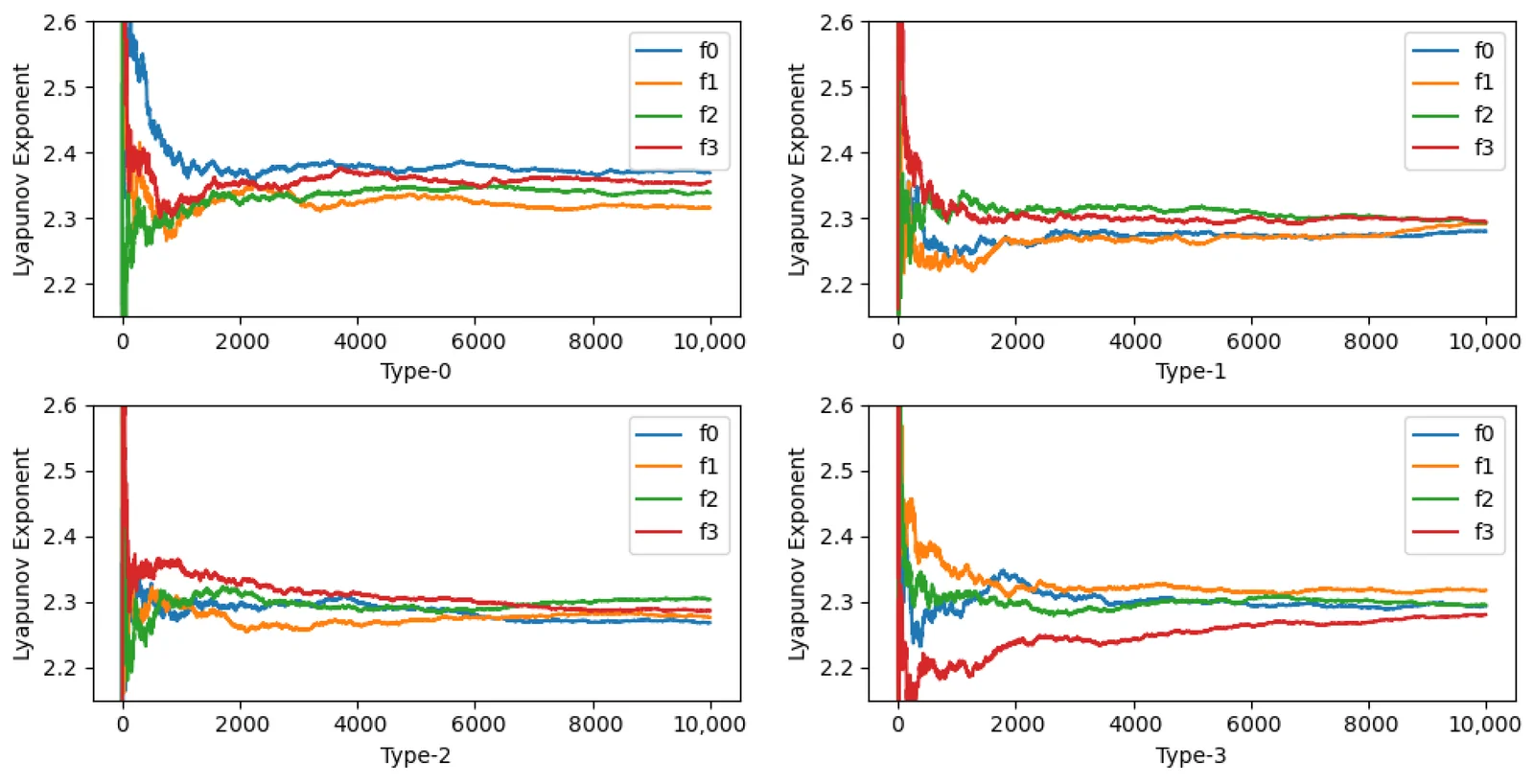
Lyapunov exponent spectrum of the four sub-chaotic maps $f_{i}$ for the four SVSC 4D chaotic system types (Type-0 to Type-3).

The Lyapunov exponent spectra of the sub-chaotic maps $f_{i}$ are presented in Figure 2. In this work, the Lyapunov exponents are evaluated using a finite-time trajectory-divergence estimator for 32-bit digital realization. Prior to the distance calculation, each 32-bit integer state variable is normalized into the real-valued domain by dividing it by 2^32^. For each test, an original trajectory is generated from a 32-bit initial state, and the perturbed trajectory is generated by flipping one bit in one 32-bit state word of the same initial condition. The two trajectories are then iterated synchronously for 10,000 iterations under the same digital chaotic map. No periodic renormalization of the perturbation is applied during the computation. For the *i*-th state component, the distance between the two trajectories is defined as

(11) $$d_{i}\left(t\right)=\left|x_{i}\left(t\right)-x_{i}^{\prime }\left(t\right)\right|,$$

and the finite-time estimate is calculated as

(12) $$\lambda_{i}\left(k\right)=\frac{1}{k}\sum_{t=1}^{k}\ln\left|\frac{d_{i}\left(t\right)}{d_{i}\left(0\right)}\right|.$$

Thus, the values in Figure 2 should be interpreted as empirical finite-time divergence estimates of the digital trajectories rather than classical Lyapunov spectra obtained from a differentiable continuous map.

All variants exhibit positive finite-time divergence estimates, indicating sensitive dependence on initial conditions. It is observed that the dynamic coupling mechanism does not significantly change the magnitude of these estimates among Type-0 to Type-3, suggesting that its primary contribution is not to increase local divergence. Instead, the structure variation introduces additional coupling diversity and improves the unpredictability of the generated states.

**Figure 3:**
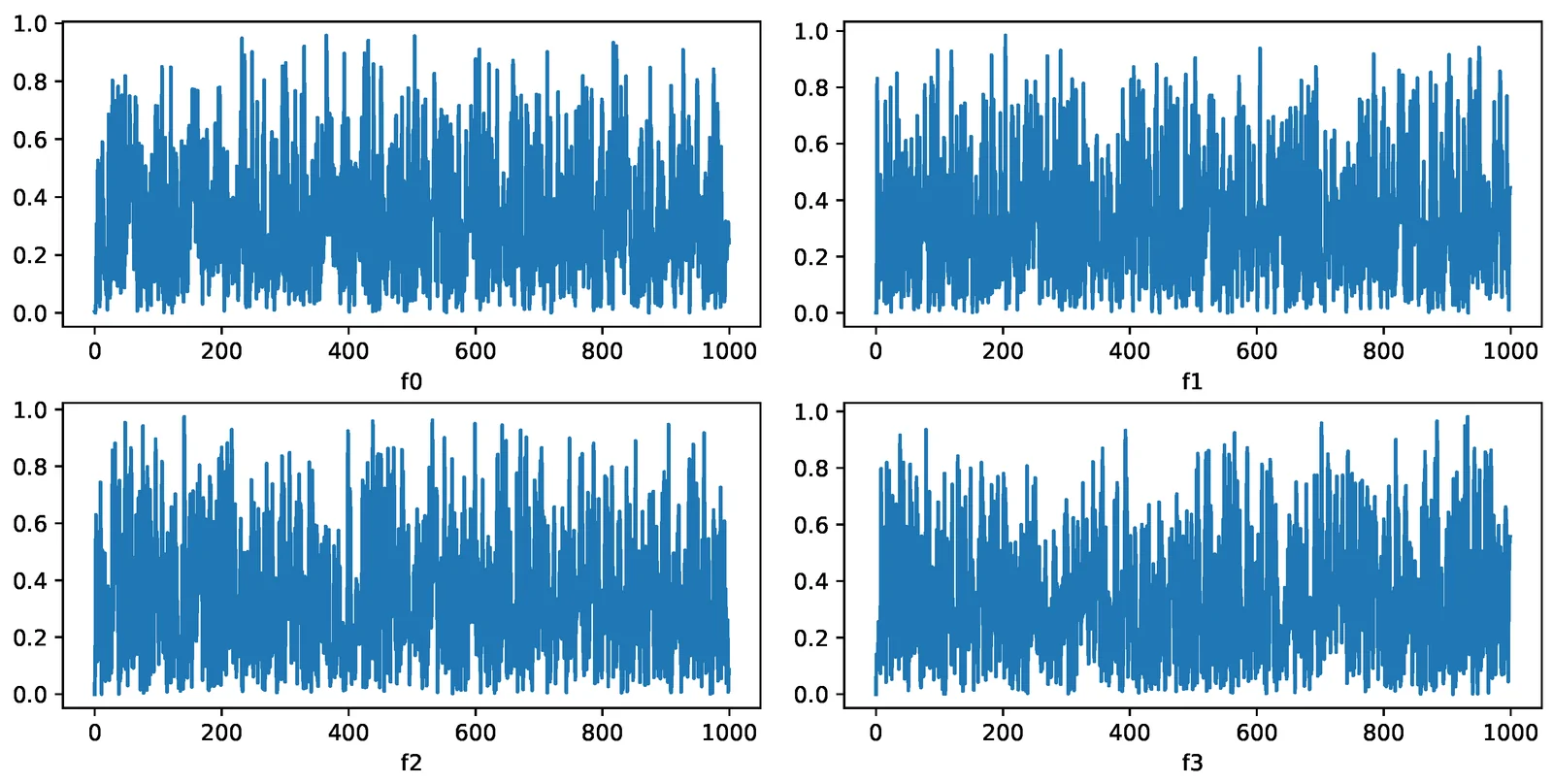
The absolute difference between $f_{i}^{j}\left(x\right)$ and $f_{i}^{j}\left(x^{\prime }\right)$ in the Type-3 SVSC 4D chaotic system under single-bit initial value perturbation.The integer states are mapped to the interval [0,1] only for visualization.

Figure 3 illustrates the absolute difference between the normalized original sub-chaotic state $f_{i}^{j}\left(x\right)$ and its perturbed counterpart $f_{i}^{j}\left(x^{\prime }\right)$ in the Type-3 SVSC 4D chaotic system, resulting from a single-bit disturbance in the initial value. It is notable that the 32-bit integer states generated by Equation (3) are mapped to the interval [0,1] only for visualization. The results show that a single-bit disturbance generates two entirely different evolutionary trajectories, indicating the system’s strong sensitivity to initial conditions. This is closely related to the avalanche effect in cryptography, that is, small input changes in the encryption algorithm will lead to huge changes in the output, which can enhance the cryptographic algorithm.

**Figure 4:**
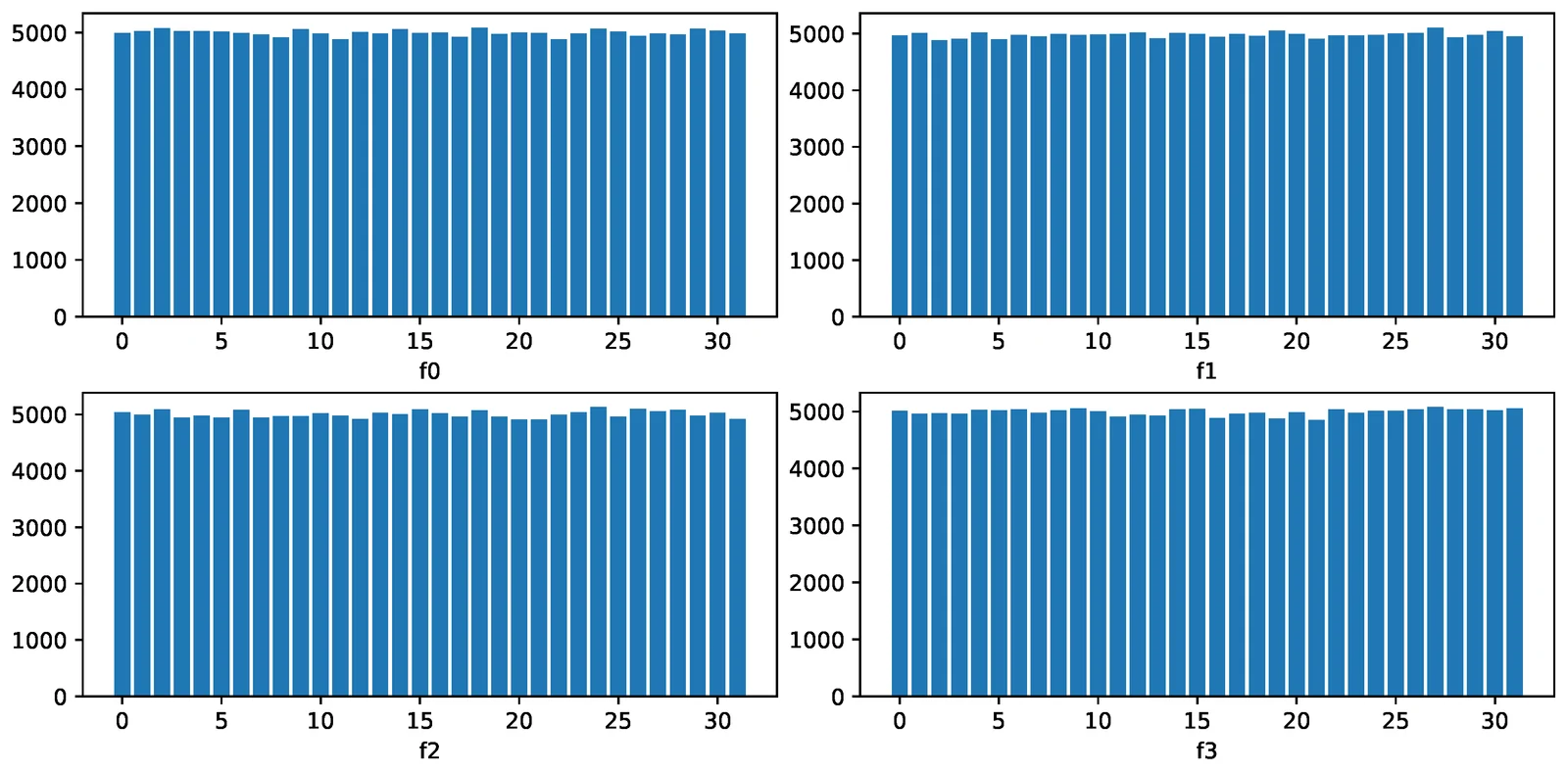
The count of 1’s occurrences per bit position in each sub-chaotic map of Type-3 SVSC 4D chaotic system.

Figure 4 illustrates the count of 1’s occurrences per bit position in each sub-chaotic map of the Type-3 SVSC 4D chaotic system. The results indicate that the chaotic states are randomly and uniformly distributed, and it is reasonable to use the bits from the states to dynamically configure the coupling structure.

Approximate entropy (ApEn) and sample entropy (SampEn) are nonlinear dynamic parameters, which are used to quantify the unpredictability and randomness of time sequences generated by chaotic systems [41]. Table 1 reports the ApEn and SampEn of the four state dimensions generated by the Type-0 to Type-3 SVSC 4D chaotic maps. The ApEn and SampEn values range from 2.1053–2.1750 and 2.1220–2.1967, respectively, indicating high complexity for all configurations. Compared with Type-0, the variable-structure maps (Type-1 to Type-3) exhibit more uniform entropy distributions across the four state dimensions, suggesting that the proposed variable-structure mechanism improves the uniformity, complexity, and unpredictability of the generated chaotic states.

Since the chaotic states are represented using 32-bit binary finite precision, the inherent properties of the chaotic system may degrade due to quantization effects and finite-state limitations. To verify that the proposed finite-precision chaotic system still maintains long-period characteristics, the chaotic cycle-finding algorithm (CCFA)  [42,43] method was employed to search and evaluate the cycle lengths of the generated chaotic sequences. Table 2 reports the periodic testing results of the proposed SVSC 4D digital chaotic systems under different precision levels. For each type and precision level, 1000 random initial states were tested with a maximum search length of 5,000,000 iterations. In addition to the average detected period, the table also reports the number of detected cycles and the minimum observed period to reveal possible short-cycle or fixed-point degradation. The symbol *U* denotes that no period was detected within the search limit.

At low precision levels, quantization suppresses the structural-control bits introduced by the dynamic mechanisms, causing Type-0 to Type-3 to degenerate into the same effective map and exhibit identical period statistics at $Q=2^{-4}$ and $Q=2^{-8}$. As the precision increases, these control bits become effective, resulting in distinct periodic behaviors among different variants. Type-2 and Type-3 show longer detected periods at $Q=2^{-10}$ and $Q=2^{-12}$ than Type-0 and Type-1. The identical Type-2 and Type-3 values at these two precision levels are caused by the truncation of the additional Type-3 bitwise-inversion control bits, making the Type-3 update equivalent to Type-2 under these quantized settings. At the actual 32-bit implementation precision, $Q=2^{-32}$, no period was detected for any of the four types within the 5,000,000-iteration search limit. These results indicate that the proposed SVSC 4D digital chaotic system does not exhibit observable short-period or fixed-point degradation under the adopted experimental bound.

## 4. DSLHash

DSLHash employs a sponge-based framework for efficient and verifiable security. Similar to state-of-the-art lightweight hashing schemes, it uses an iterated SPN structure [10,11,12]. Design details, including parameter settings and security assertions, are tabulated in Table 3.

**Figure 5:**
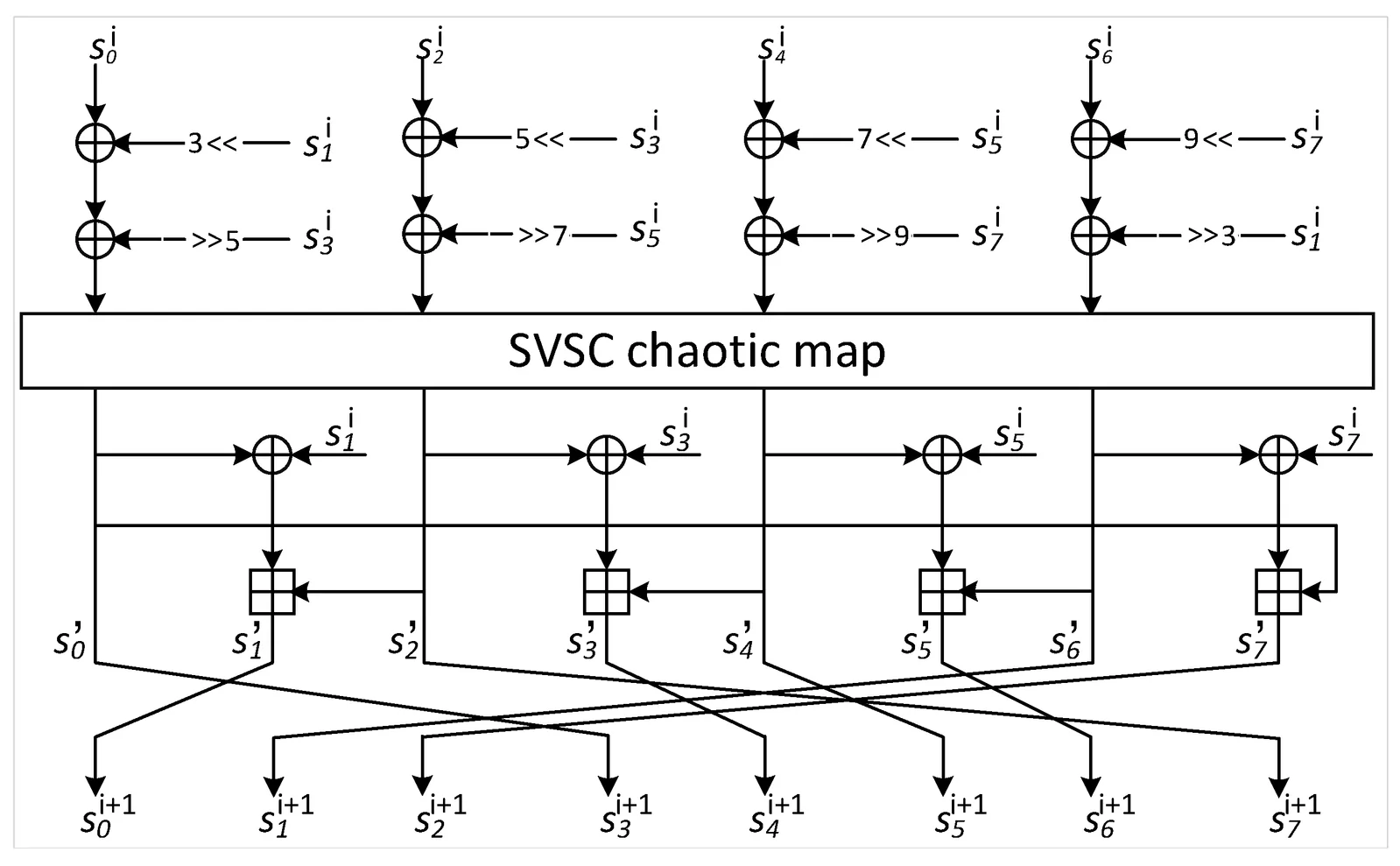
Structure of the proposed permutation function.

To achieve desirable cryptographic properties with efficient diffusion characteristics, DSLHash incorporates two key components: (1) a dynamic substitution layer instantiated using a SVSC 4D Chaotic map to enhance nonlinear diffusion, and (2) a Type-II GFS-inspired permutation layer [27,44] employing lightweight transformations, which enhances diffusion efficiency through structured word-level shuffling. A detailed schematic of the permutation function is presented in Figure 5.

In this version, the sponge state *S* is set to 256 bits in order to reduce the hardware implementation overhead. This state size is smaller than those of the other NIST LWC finalists listed in the table, except for PHOTON-Beetle. To achieve a balance between security and absorption rate, the rate r is set to 64 bits. The number of rounds is set to 12, which is consistent with the five representative lightweight hash functions selected for comparison. In addition, reduced-round analyzes are conducted in Section 5.3 and  Section 5.4 to evaluate the diffusion performance and security margin of the proposed construction. With a state size of *S* = 256 bits and a rate of *r* = 64 bits, the capacity is c=S−r=192 bits. According to the generic security bounds of sponge constructions, the collision resistance is approximately $2^{c/2}=2^{96}$, and the second-preimage resistance is of the same order.

Although the proposed hash function targets a relatively lower security level, this design choice enables a significant reduction in hardware complexity and resource consumption, making it more suitable for resource-constrained IoT applications. It is worth noting that the proposed construction methodology is not inherently limited to the parameter set listed in Table 3. By increasing the internal state size and capacity, the same design framework can be directly extended to provide higher collision resistance and stronger security levels, making it suitable for applications with more stringent security requirements.

As depicted in Figure 5, DSLHash’s permutations operate on a 256-bit internal state through multiple iterative transformation rounds. The state is partitioned into (256/r)*r*-bit words. The first word serves as the outer part (bit-rate portion) and the remaining (256/r−1) words constitute the inner capacity section. The overall algorithm comprises three principal phases: initialization, absorbing, and squeezing.

In the initialization phase, the input message undergoes standardized padding and is partitioned into consecutive *r*-bit blocks. Subsequently, during the absorbing phase, each message block is processed through iterative applications of the permutation function. This phase continues until all message blocks have been fully absorbed into the state. In the squeezing phase, the permutation function is executed in a staggered manner, with *r*-bit segments extracted sequentially from the state to form the final hash output. Detailed implementations of these phases are provided in the dedicated subsections below.

### 4.1. Initialization Phase

In the initialization phase, the internal state is initialized by the initialization vector (IV), which is fixed as a constant value, as illustrated in Algorithm 2. Then the input message M undergoes standardized padding to ensure its length is a multiple of *r* bits. Following the widely employed padding scheme in symmetric cryptography [10,11,12], Specifically, a delimiter byte 0x80 is appended to the input message, followed by the minimum number of zero bytes required to make the padded message length a multiple of 8 bytes. The processed message is subsequently partitioned into contiguous *r*-bit chunks ${\left\{m_{i}\right\}}_{i=1}^{L}$, forming the input sequence for the initialization procedure.

A formal algorithmic description of this phase is provided in Algorithm 2.
**Algorithm 2:** Message initialization and padding**Data**: Input message *M* (8-bit byte array)**Result**: Padded message *m*
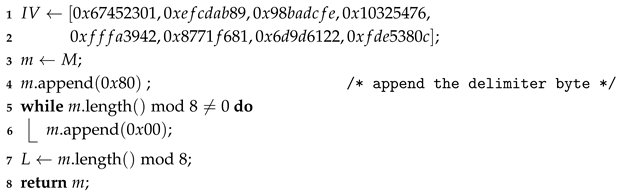


### 4.2. Absorbing Phase

The absorbing phase represents the most critical component of DSLHash, as it accomplishes both information compression and maximal diffusion of the input message blocks.

Each incoming message block undergoes a series of structured transformations: first, it is XORed with the *r*-bit outer portion of the internal state (controlling the bitrate), followed by iterative applications of the permutation function *P*. This process is mathematically formalized as

(13) $$S\leftarrow P^{12}(\left(S_{r}⊕m_{i}\right)\left|\right|S_{c}),$$

demonstrating the systematic integration of message blocks into the state. Upon the complete absorption of all message blocks, the evolved state serves as the basis for the subsequent squeezing phase.

The design of the permutation function, as shown in Figure 5, is inspired by the Type-II GFS [27,44]. The first step of the permutation is dividing the XOR-ed internal state into eight 32-bit sub-blocks, $\{s_{0},s_{1},$$\ldots s_{7}\}$. The odd-indexed state words $s_{i}$ (i={1,3,5,7}) are independently rotated by 3, 5, 7, and 9 bits, respectively. The rotation directions are alternately selected as left and right rotations for adjacent odd-indexed words. The even-indexed blocks are then XORed with the adjacent rotated odd-indexed blocks and fed into the SVSC 4D chaotic map as the initial states of its four 1D chaotic maps. The resulting status of each 1D chaotic map is used as the even temporary internal sub-state $s_{i}^{\prime }$ (i={0,2,4,6}). The odd-indexed temporary internal states $\left\{s_{1}^{\prime },s_{3}^{\prime },s_{5}^{\prime },s_{7}^{\prime }\right\}$ are generated through a two-stage transformation process: (1) A 32-bit modular addition operation with its preceding temporary state $s_{(i-1)mod\;8}^{\prime }$ in a cyclic manner; (2) a bitwise XOR with its succeeding temporary state $s_{(i+1)mod\;8}^{\prime }$. This dual-operation structure ensures that input information undergoes both linear (modular addition) and nonlinear (XOR) transformations, effectively propagating the initial entropy into the odd-indexed sub-states. Subsequently, an eight-packet shuffle operation [27,44] is employed to generate the final internal state defined as

(14) $$\left(s_{0}^{i+1},s_{1}^{i+1},s_{2}^{i+1},s_{3}^{i+1},s_{4}^{i+1},s_{5}^{i+1},s_{6}^{i+1},s_{7}^{i+1}\right)=\left(s_{1}^{\prime },s_{6}^{\prime },s_{7}^{\prime },s_{0}^{\prime },s_{3}^{\prime },s_{4}^{\prime },s_{5}^{\prime },s_{2}^{\prime }\right).$$

### 4.3. Squeezing Phase

The hash output is generated by iteratively extracting the outermost *r*-bit segment from the current state until the desired output length *n* is achieved through $t=\left\lceil n/r\right\rceil$ processing blocks. Following each extraction phase, the internal state undergoes a 12-stage permutation process denoted as $P^{12}$.

## 5. Security Evaluation

In this section, we conduct a security analysis of the proposed hash function in the following aspects: diffusion and confusion analysis, collision resistance, differential approximation probability, and theoretical security proof. In this section, the proposed hash function is implemented in C programming language, and the experiments were performed on a machine with a 2.59 GHz Intel Core i7-9750H and 16 G of installed RAM. To validate the effectiveness of the hash function constructed using the SVSC 4D chaotic system with 32-bit finite precision, we compare DSLHash0–DSLHash3—implementations featuring different dynamic substitution configurations—against five NIST LWHFs employing static substitution. Three real-valued chaotic hash functions are also evaluated. Here, DSLHashX represents hash function with Type-X chaotic map.

### 5.1. Hash Results of Messages

In the first experiment, we perform a qualitative experiment to depict the diffusion and confusion property of our proposed hash function. Assume that the original message is “*A hash function is any function that can be used to map data of arbitrary size to fixed-size values, though there are some hash functions that support variable-length output. The values returned by a hash function are called hash values, hash codes, hash digests, digests, or simply hashes*”, the hash value is generated and then stored. Then, single-bit perturbations are introduced to the original message, and all the corresponding hash values are generated. The hash values for the original and modified messages are shown in Table 4. It can be observed that the proposed hash function produces entirely different hash values for each message variant. This indicates that our proposed hash function is highly sensitive to slight changes in the input message. The sensitivity will be further studied in the following sections.

### 5.2. Statistic Analysis of Diffusion and Confusion

**Figure 6:**
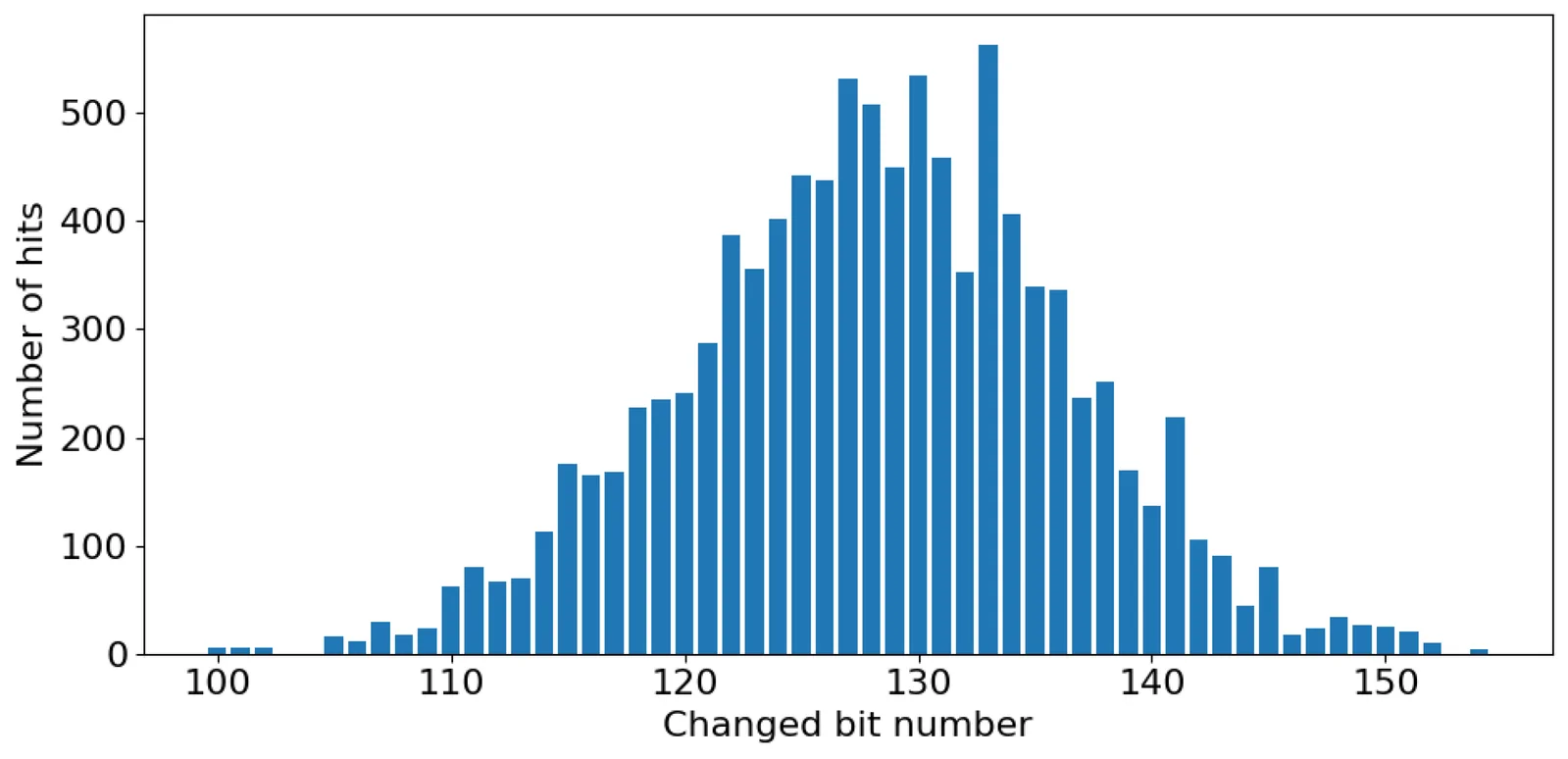
Distribution histogram of $X_{i}$.

To statistically analyze the diffusion and confusion properties of the proposed hash function, we conduct the following statistical tests: A paragraph of message is randomly chosen and its hash value is generated, then a new message is obtained by toggling a random bit within the original message, and a new hash value is subsequently generated. These two hash values are compared and the total number of changed bits is denoted as $X_{i}$, where *i* represents the number of changed bits. The above test is performed *N* times, and the corresponding distribution of $X_{i}$ is shown in Figure 6, where N= 10,000. As shown in Figure 6, the changed bit number $X_{i}$ is evenly and normally distributed, and centers around the number-128 bit. Similar to previous works [24,44], we can calculate the following statistics by using $X_{i}$.

Mean changed bit number:

(15) $$\bar{X}=\frac{1}{N}\sum_{i=1}^{N}X_{i}.$$

Mean changed probability:

(16) $$P=\frac{\bar{X}}{256}\times 100\%.$$

Standard variance of changed bit number:

(17) $$\Delta X=\sqrt{\frac{1}{N-1}\sum_{i=1}^{N}{\left(X_{i}-\bar{X}\right)}^{2}}.$$

Standard variance:

(18) $$\Delta P=\sqrt{\frac{1}{N-1}\sum_{i=1}^{N}{\left(\frac{X_{i}}{256}-P\right)}^{2}}\cdot 100\%.$$

Here, ΔX and ΔP represent the stability of diffusion and confusion respectively. For an ideal diffusion effect, a small change in the input message should result in 128-bit changed bits, and the ideal *P* should be 50%. Table 5 lists the test results with N= 256, 512, 1024, 2048, 4096, and 10,000. It can be seen that both $\bar{X}$ and *P* are very close to the ideal value 128-bit and 50%, and ΔX and ΔP are very small. These statistical results show that the capabilities of diffusion and confusion of the proposed hash function are strong and stable. The diffusion and confusion characteristics for N= 10,000 are compared against five NIST finalists [28] in Table 6. The results indicate that the proposed hash function achieves comparable diffusion and confusion properties to those of the selected NIST finalists and real-valued chaotic hash functions.

### 5.3. Analysis of Collision Resistance

As shown in Table 3, the proposed hash function is highly collision resistant due to the use of the sponge construction. To illustrate this property quantitatively, we perform the following experiments: First, a pair of messages and their corresponding hash values (denoted by *H* and $H^{\prime }$ respectively) are generated similarly to the test in Section 5.2. Instead of comparing each bit of the hash values, the two *n*-bit hash values are partitioned into nb sub-blocks with *k*-bit length. The number of sub-blocks that have the same value at the same location in two hash values is referred to as the number of hits *w*, which can be represented as

(19) $$w=\sum_{i=1}^{nb}f\left(t\left(e_{i}\right),t\left(e_{i}^{\prime }\right)\right),f\left(x,y\right)=\left\{\begin{array}{cc} 1 & :\;x=y\\ 0 & :\;x\neq y \end{array}\right.,$$

and the absolute difference of two hash values is calculated as

(20) $$d=\sum_{i=1}^{nb}\left|t\left(e_{i}\right)-t\left(e_{i}^{\prime }\right)\right|,$$

where the function t(·) converts the sub-block to their equivalent decimal values. Obviously, smaller *w* or bigger *d* indicates the stronger collision resistance of the hash function. Assuming the hash value is uniformly and randomly distributed, the theoretical number of *w* with different values during *N* independent experiments can be expressed as

(21) $$\begin{array}{cc} W_{N}\left(w\right) & =N\times Prob\{w\}\\ \; & =N\frac{nb!}{w!(nb-w)!}{\left(\frac{1}{2^{k}}\right)}^{w}{\left(1-\frac{1}{2^{k}}\right)}^{nb-w}, \end{array}$$

where w=0,1,…,nb, and a collision will happen if w=nb=n/k. In our experiments, *k* is set with 8 and nb=n/k=32, and the collision test is performed N= 10,000 times. The theoretical values are $W_{N}\left(0\right)=8822.2$, $W_{N}\left(1\right)=1107.8$, $W_{N}\left(2\right)=67.3$, $W_{N}\left(3\right)=2.6$. As shown in Table 7, the results of our proposed hash function are $W_{N}\left(0\right)=8880$, $W_{N}\left(1\right)=1048$, $W_{N}\left(2\right)=72$, $W_{N}$ (>2) = 0, which coincide very well with the theoretical values and the results of the five finalists of the NIST LWC and the real-valued chaotic hash functions. The hash functions with different types of chaotic maps perform good collision resistance characteristics too.

To further study the collision resistance characteristic of the proposed dynamic substitution layer, we test the number of hits when the permutation functions consist of only one round. As depicted in Table 8, the results of DSLHash still coincide well with the theoretical values, and exceed PHOTON, ASCON and Xoodyak. Furthermore, the hash function based on the Type-2 chaotic map demonstrates stronger collision resistance compared to those utilizing other types of chaotic maps.

According to Refs. [47,48], the uniform distribution mean value is $\frac{255\times 32}{2}=4080$, and the theoretical mean absolute difference is $\frac{2\times 4080}{3}=2720$ for a 256-bit hash. For N= 10,000, the minimum, maximum, and mean absolute difference are illustrated in Table 9, and it is indicated that the mean absolute difference is extremely near to the theoretical value. The above results indicate that the proposed algorithm has strong collision resistance.

### 5.4. Differential Approximation Probability (DAP)

DAP is an important metric for evaluating the resistance of cryptographic primitives against differential cryptanalysis. It describes the probability that a specific input difference propagates to a particular output difference after applying a nonlinear transformation. Ideally, a secure cryptographic component should exhibit a uniform differential distribution, where all nonzero input differences lead to output differences with approximately equal probabilities [14,49] defined as

(22) $$DP_{f}=\max_{\begin{array}{c} \Delta x\neq 0,\Delta y \end{array}}\left(\frac{\#\left\{x\in X|f(x)⊕f(x⊕\Delta x)=\Delta y\right\}}{2^{m}}\right).$$

As shown in Equation (22), for a given permutation function *f*, the DAP is defined as the ratio between the number of input pairs (x,x⊕Δx) producing a certain output difference Δy and the total number of possible input pairs with the specified input difference Δx. Here, *X* is the set of all possible input values and $2^{m}$ is the number of its elements. A lower maximum DAP indicates a smaller probability of predictable differential propagation, implying stronger diffusion and resistance against differential attacks.

To evaluate the differential characteristics of the proposed permutation function, 45 input-difference test cases were constructed. Each test case was independently executed 100,000 times to obtain statistically reliable results. Table 10 summarizes the differential characteristics of the reduced-round DSLHash0–DSLHash3 and compares them with those of PHOTON-Beetle, which shares the same 256-bit state.

It is illustrated that the DAP of DSLHash0 is higher than DSLHash1–DSLHash3 and the DAPs of DSLHash is lower than PHOTON-Beetle. DSLHash0 and PHOTON-Beetle correspond to static permutation functions without the complete dynamic substitution and state variation mechanisms employed in DSLHash1–DSLHash3. Therefore, the relatively higher DAPs indicate weaker differential dispersion in these configuration. After introducing the dynamic substitution layers and structure variation mechanisms, DSLHash1–DSLHash3 exhibit significantly reduced DAP values, demonstrating the effectiveness of these components in disrupting differential propagation. Furthermore, the multi-round differential propagation characteristics of DSLHash0–DSLHash3 were also investigated. For all tested input differences, no repetitive output-difference propagation patterns were observed after two rounds, indicating that the proposed permutation function can effectively disrupt differential propagation and provide desirable diffusion properties against differential cryptanalysis. Although the diffusion capability reaches a stable level after a small number of rounds, 12 rounds are adopted as a conservative design choice to provide an additional security margin against potential structural weaknesses and to ensure sufficient state mixing.

### 5.5. Randomness Test Using NIST SP 800-22

Ideally, the output digest of a cryptographic hash function should exhibit pseudorandom behavior, making it computationally indistinguishable from a truly random binary sequence. High randomness is essential for ensuring unpredictability and preventing statistical attacks. To evaluate the statistical randomness of the hash digests generated by the proposed DSLHash, the NIST SP 800-22 Rev. 1a Statistical Test Suite was employed [50]. Table 11 summarizes the NIST SP 800-22 test results for the binary sequences generated by the proposed hash function. The tested sequences were generated from the Type-3 DSLHash output. For each subsequence index *s*, DSLHash was repeatedly evaluated on indexed messages of the form DSLHash-NIST ‖ s ‖ j, where *j* denotes the digest counter. Each hash evaluation produces a 256-bit digest, and consecutive digests were concatenated until the required subsequence length was reached. In total, 1000 binary subsequences were generated, each containing 1,000,000 bits; therefore, the total amount of tested data was $10^{9}$ bits, corresponding to 125 MB. The NIST STS program was run with a sequence length of 1,000,000 bits and 1000 input sequences at a significance level of α=0.01.

As shown in Table 11, all statistical tests were successfully passed, with *p*-values ranging from 0.02623 to 0.99668. Most tests achieved pass rates above 986/1000, while the Random Excursions and Random Excursions Variant tests used an effective sample size of 635 and achieved 629/635, satisfying the NIST acceptance criteria. These results demonstrate that the generated hash sequences possess good statistical randomness and unpredictability.

### 5.6. Theoretical Security Proof

The above statistical results show that the proposed hash function can achieve near-ideal statistical results in terms of diffusion, confusion, collision resistance, and hash value distribution. These statistical results demonstrate uniform and independent distribution characteristics, providing strong evidence that the proposed dynamic substitution function *P* effectively functions as a random sponge function. Building on this foundation, we provide a theoretical security analysis of the proposed hash function against collision, preimage, and second-preimage attacks, assuming a sponge construction instantiated with a random permutation.

**Collision resistance**. Collision resistance appears to be one of the most important properties of cryptographic hash functions. A state collision can be easily produced from an inner collision. Given two messages $M_{1}\neq M_{2}$ with $P\left(M_{1}\right)=P\left(M_{2}\right)$, one can produce a state collision on $M_{1}\left|\right|A\neq M_{2}\left|\right|B$ for any *A*, *B* that satisfies $P\left(M_{1}\right)⊕A=P\left(M_{2}\right)⊕B$. As our dynamic substitution function has n>c, the best strategy to generate an output collision is to use an inner collision. In a random sponge, the complexity of generating an inner collision is of the order $2^{c/2}=2^{96}$ [33,51].

**Pre-image resistance**. In a sponge, a pre-image can be obtained by binding an output string to a state *s* and subsequently finding a path to that state. As the structure of the substitution function depends on its previous state, attackers cannot compute a state $t=P^{-1}\left(s\right)$. Therefore, after having bound the output to a state *s*, attackers need to guess a state. The complexity for finding a pre-image for a sponge function with a random permutation is hence $2^{n-r}+2^{c/2}$ for n<s. As our dynamic substitution function has n=s, the complexity of pre-image attacks is $2^{256-64}+2^{192/2}$. Thus, it is reasonable to claim that the proposed design provides pre-image resistance with an attack complexity of at least $2^{192}$.

**Second pre-image resistance**. In a sponge function, attackers could have a second pre-image if they could find a second path to the inner state. The cost function for a random T-sponge is of the order $2^{c}/l$ if $l<2^{c/2}$ [33,51]. Assuming the maximum length $l_{max}$ is limited to $2^{c/2}=2^{96}$, the complexity of finding a second path to the inner state is above the order $2^{c}/l_{max}=2^{c/2}=2^{96}$. The above theoretical analysis results are summarised in Table 3.

## 6. Hardware Implementation

Our DSLHash implementation builds upon the NIST LWC hardware reference design for Ascon v1.2 [52,53], enabling direct comparison with other lightweight hash functions through consistent evaluation metrics and testing environments. The dynamic substitution layer was realized using VHDL, and synthesized using Xilinx Vivado Design Suite 2023.1 on Artix-7 (xc7a12tcsg325-3) FPGA devices. We perform a comprehensive comparative analysis against all five final-round NIST LWC hash function candidates, evaluating four key hardware performance metrics: resource utilization, maximum operating frequency, energy efficiency, latency, and throughput.

The hardware implementation results of DSLHash are presented in Table 12. The evaluation results demonstrate that our lightweight hash function maintains competitive resource efficiency comparable to state-of-the-art NIST LWC finalists, while incorporating different types of dynamic substitution layers. Notably, frequency analysis reveals our design operates at 91 MHz versus the 135–225 MHz range of LWC finalists, as the dynamic substitution layer introduces additional critical path delays that limit clock performance. The utilization of look-up tables (LUTs) is approximately 30% below the threshold of 2520 LUTs, while flip-flop (FF) usage is significantly lower than that of the reference designs. This substantial margin enables us to strategically allocate additional LUT and FF resources to potentially achieve higher operational speed.

Using Xilinx’s Vivado Design Suite 2023.1 power analysis tool, we assess the power efficiency of our DSLHash implementations and benchmark them against the reference LWC hash functions. All power measurements are conducted at an operating frequency of 75 MHz. Switching Activity Interchange Format (SAIF) files are generated by simulating the hash functions with 20 distinct 16-byte input messages [52]. The power consumption and energy-per-bit are evaluated, with detailed results summarized in Table 13. The results indicate that the dynamic coupling structure from Type-1 to Type-3 slightly increases the power and energy-per-bit, and the energy consumption is comparable to the reference hash functions.

Since a hash function’s throughput is input-dependent, we evaluate it using the following input sizes: 16 bytes, 64 bytes, 1536 bytes, and long blocks [52]. The throughput for given input sizes is computed as

(23) $$TP\left(Mbps\right)=\frac{Number\;of\;bits\cdot Freq.}{Clock\;Cycles}.$$

For long blocks, the clock cycle count is determined by the execution time difference between N+d and *N* blocks, where N=4 and d=2. The corresponding bit count is computed as d·Block_size·8, with Block_size=1536. The evaluation results are illustrated in Table 14. Due to DSLHash’s lower operating frequency, its throughput rate is comparatively lower than the reference hash functions. Although DSLHash0 and DSLHash1 achieve lower hardware cost and higher throughput, their simpler structures result in shorter cycle behaviors, which may affect their security margins. DSLHash2 and DSLHash3 introduce stronger dynamic coupling mechanisms and provide improved security characteristics with additional hardware overhead. Therefore, the choice of configuration depends on the application requirements. We can improve their throughput by optimizing the design to achieve higher frequency. These research objectives will be implemented in our future work.

## 7. Conclusions

This paper presents a lightweight hash function architecture that integrates a dynamic substitution layer with a structure-varying chaotic system. The main contributions include three aspects. First, a practical multiplier-free implementation of the SVSC 4D chaotic map is proposed using lightweight arithmetic and logical operations, enabling efficient finite-precision hardware realization. Second, a dynamic substitution layer is introduced within a generalized Feistel-based permutation framework to enhance state transition diversity and improve diffusion characteristics. Third, the proposed hash function is constructed using a sponge framework, and comprehensive security and hardware evaluations are conducted. The experimental results demonstrate that DSLHash achieves desirable statistical randomness, diffusion performance, and differential propagation characteristics while maintaining a compact hardware implementation. The proposed design provides a flexible lightweight hash construction methodology that balances security characteristics, hardware complexity, and implementation cost, making it suitable for resource-constrained applications. Although the dynamic substitution layer of DSLHash2 and DSLHash3 introduces additional critical-path delays and results in a trade-off between security enhancement and operating frequency/throughput, it provides improved structural diversity and security-oriented properties. Future work will focus on optimizing the substitution and permutation architectures to further improve throughput and frequency while preserving the lightweight characteristics of the proposed design.

## Figures and Tables

**Table 1:** ApEn and SampEn of the four state dimensions generated by the Type-0 to Type-3 SVSC 4D chaotic maps.

Entropy	Type	$x^{0}$	$x^{1}$	$x^{2}$	$x^{3}$
ApEn	Type-0	2.1052603	2.1749578	2.1615579	2.1617243
ApEn	Type-1	2.1594681	2.1634646	2.1683689	2.1698758
ApEn	Type-2	2.1625056	2.1635315	2.1670125	2.1674488
ApEn	Type-3	2.1584556	2.1555065	2.1677737	2.1659376
SampEn	Type-0	2.1219540	2.1966825	2.1815794	2.1809046
SampEn	Type-1	2.1817754	2.1847279	2.1899719	2.1923895
SampEn	Type-2	2.1859815	2.1867989	2.1884691	2.1912321
SampEn	Type-3	2.1788609	2.1772604	2.1890797	2.1855491

**Table 2:** Periodic testing results of the proposed SVSC 4D digital chaotic systems.

Type	Metric	$2^{-4}$	$2^{-8}$	$2^{-10}$	$2^{-12}$	$2^{-15}$	$2^{-32}$
Tp0	Avg	59.63	5147.39	98,670.25	403,224.94	1,170,158.20	U
	Min	1	126	1624	3216	649,376	–
	Detected	1000/1000	1000/1000	1000/1000	1000/1000	5/1000	0/1000
Tp1	Avg	59.63	5147.39	99,330.25	439,568.03	U	U
	Min	1	126	680	180,153	–	–
	Detected	1000/1000	1000/1000	1000/1000	472/1000	0/1000	0/1000
Tp2	Avg	59.63	5147.39	136,818.11	1,665,463.00	U	U
	Min	1	126	36,335	1,665,463	–	–
	Detected	1000/1000	1000/1000	1000/1000	68/1000	0/1000	0/1000
Tp3	Avg	59.63	5147.39	136,818.11	1,665,463.00	U	U
	Min	1	126	36,335	1,665,463	–	–
	Detected	1000/1000	1000/1000	1000/1000	68/1000	0/1000	0/1000

U represents no detected period within the maximum search length of 5,000,000 iterations. For each type and precision level, 1000 random initial states were tested. Avg. period and Min. period are calculated from detected cycles only; “–” indicates that no cycle was detected.

**Table 3:** The parameters and security assertions of DSLHash.

	n (bit)	s (bit)	r (bit)	R Number Round	Security (bit)		
					Preimage	2nd Preimage	Collision
PHOTON-Beetle	256	256	32	12	224	112	112
ASCON-HASH	256	320	64	12	192	128	128
ESCH256	256	384	128	12	128	128	128
Xoodyak	256	384	128	12	128	128	128
Gimli	256	384	128	12	128	128	128
DSLHash	256	256	64	12	192	96	96

**Table 4:** Message variants and their corresponding hash values.

	Message Variant	Hash Value
1	Original message	8611f5e0db36ead174683caab1a31691
		58ea474d2edaa9e30f014f62c0e1b6a3
2	Add a whitespace at the beginning of the message	facf1b0900648e9b8cc7254a92ae3f14
		2d89ce2a6736d6fd2dd955e8bc99bcb4
3	Change first character to lowercase	c42f1b13d746b258dc10860685af9601
		40520d4533575f75087ad77acd83c5f9
4	Change ‘.’ after output to ‘,’	b5fc82fef8a4036aa724cb72bf7665c4
		e820b7f6d3b62d7c3766ee3a28fd40d6
5	Add an extra whitespace between ‘simply’ and ’hashes’	d95a201c7340daed06f15310dd14ca89
		074732449a19a8c620ede7999d1ba220
6	Change first character in ‘though’ to uppercase	4c5e6fa7a274e86e9c267f04b9336e97
		dcac7c8cc9958417b26158c8ceb55a2c
7	Change last character ‘.’ to ‘?’	06f4280359db7229b3a4b5bad305d1d6
		24d1f291f973af8b6a5912e6334127cc

**Table 5:** Statistical results of confusion and diffusion for 256-bit hash values.

	*N* = 256	*N* = 512	*N* = 1024	*N* = 2048	*N* = 4096	*N* = 10,000	Mean
$\bar{X}$	128.9023	128.7187	128.2695	128.2275	128.3057	128.2318	128.4569
*P*	0.5035	0.5028	0.5011	0.5009	0.5012	0.5009	0.5017
ΔX	8.2824	7.8145	8.1365	8.1133	8.2419	8.2413	8.1383
ΔP(%)	3.2353	3.0526	3.1783	3.1693	3.2195	3.2193	3.1791
$X_{min}$	107	107	100	100	100	100	102
$X_{max}$	152	152	152	154	154	154	153

**Table 6:** Comparison of diffusion and confusion characteristics for N= 10,000.

	DSLHash0	DSLHash1	DSLHash2	DSLHash3	PHOTON-Beetle	ASCON-HASH	ESCH256	Xoodyak	Gimli	Ref. [43]	Ref. [45]	Ref. [46]
$\bar{X}$	127.8712	128.0206	127.5966	128.2318	127.8826	127.5464	127.9632	127.7436	128.0644	127.8563	128.0449	127.8620
*P*	0.4995	0.5000	0.4984	0.5009	0.4995	0.4982	0.4998	0.4989	0.5003	0.4994	0.5001	0.4995
ΔX	7.8937	7.6858	8.1374	8.2413	8.1400	8.0182	7.9268	7.8155	8.0255	8.0326	8.0900	8.0504
ΔP(%)	3.0835	3.0023	3.1787	3.2193	3.1797	3.1321	3.1106	3.2909	3.1598	3.1378	3.1602	3.1447
$X_{min}$	102	104	104	100	103	84	104	102	103	100	100	102
$X_{max}$	156	153	156	154	158	152	157	155	158	161	156	155

**Table 7:** Number of hits for N= 10,000.

Hits	0	1	2	3	4	5
Ideal	8822.2	1107.8	67.3	2.6	0	0
PHOTON	8879	1040	81	0	0	0
ASCON	8773	1169	58	0	0	0
ESCH256	8754	1194	52	0	0	0
Xoodyak	8653	1259	81	7	0	0
Gimli	8821	1104	69	6	0	0
Ref. [43]	8766	1162	71	0	0	0
Ref. [45]	8851	1087	59	3	0	0
Ref. [46]	8816	1109	73	2	0	0
DSLHash0	8871	1049	80	0	0	0
DSLHash1	8802	1132	64	2	0	0
DSLHash2	8814	1102	84	0	0	0
DSLHash3	8880	1048	72	0	0	0

**Table 8:** Comparison of collision resistance characteristic when R=1.

Hits	0	1	2	3	4	5	≥6
Ideal	8822.2	1107.8	67.3	2.6	0	0	0
PHOTON	8802	947	6	0	0	0	172
ASCON	8572	1189	96	89	37	14	3
ESCH256	8756	1204	40	0	0	0	0
Xoodyak	7795	1152	255	91	51	12	1796
Gimli	8869	1090	41	0	0	0	0
DSLHash0	8858	1079	56	4	3	0	0
DSLHash1	8819	1113	50	18	0	0	0
DSLHash2	8811	1129	60	0	0	0	0
DSLHash3	8782	1120	96	2	0	0	0

**Table 9:** Comparison of absolute difference *d* for N= 10,000.

	Max	Min	Mean
Ideal	–	–	2720
PHOTON-Beetle	3692	1488	2616
ASCON-HASH	4205	1565	2727
ESCH256	3947	1654	2834
Xoodyak	4004	1713	2756
Gimli	3616	1597	2584
Ref. [43]	3940	1408	2733
Ref. [45]	3863	1607	2738
Ref. [46]	4285	1794	2995
DSLHash0	3994	1727	2707
DSLHash1	3745	1806	2739
DSLHash2	3951	1742	2706
DSLHash3	3643	1598	2619

**Table 10:** DAPs of reduced-round DSLHash0–DSLHash3 and comparison with PHOTON-Beetle.

	DSLHash0	DSLHash1	DSLHash2	DSLHash3	PHOTON-Beetle
Round 1	9.496%	2.704%	2.784%	2.686%	49.971%
Round 2	0.001%	0.001%	0.001%	0.001%	0.005%
Round 3	0.001%	0.001%	0.001%	0.001%	0.001%
Round 4	0.001%	0.001%	0.001%	0.001%	0.001%

**Table 11:** NIST SP 800-22 test results.

Test Index	*p*-Value	Proportion	Result
Frequency	0.70342	993/1000	Success
Block Frequency	0.02623	988/1000	Success
Cumulative Sums *	0.84795	991/1000	Success
Runs	0.59348	993/1000	Success
Longest Run	0.91403	991/1000	Success
Rank	0.45030	994/1000	Success
FFT	0.71364	991/1000	Success
Non-Overlapping Template *	0.44964	989/1000	Success
Overlapping Template	0.69314	988/1000	Success
Universal	0.24675	987/1000	Success
Approximate Entropy	0.72987	992/1000	Success
Random Excursions *	0.53119	629/635	Success
Random Excursions Variant *	0.55731	629/635	Success
Serial *	0.45535	986/1000	Success
Linear Complexity	0.99668	986/1000	Success

* average result of multiple tests is shown.

**Table 12:** Resource usage and maximum frequency of DSLHash and comparison with five lightweight hash functions from the finalists of the NIST LWC [54].

Technique	LUTs	FFs	Slices	Freq. (MHz)
Ascon1a_v1 [53]	2232	487	592	189
Ascon1b [55]	1832	474	481	213
Xoodoo_R2 [56]	1268	411	348	206
PHOTON-Beetle [57]	1001	608	313	225
SPARKLE [58]	3548	1473	991	135
DSLHash0	1211	282	343	115
DSLHash1	1374	285	395	101
DSLHash2	1611	285	444	91
DSLHash3	1757	286	485	91

**Table 13:** Energy-per-bit for hashing of 16-Byte messages at 75MHz and comparison with five lightweight hash functions from the finalists of the NIST LWC [54].

	Cycles	Power (W)	Energy-per-Bit (nJ/bit)
Ascon1a_v1 [53]	1992	0.095	0.986
Ascon1b [55]	2016	0.093	0.977
Xoodoo_R2 [56]	793	0.090	0.372
PHOTON-Beetle [57]	1311	0.114	0.779
SPARKLE [58]	1686	0.122	1.072
DSLHash0	1992	0.085	0.882
DSLHash1	1992	0.092	0.955
DSLHash2	1992	0.095	0.986
DSLHash3	1992	0.097	1.007

**Table 14:** The latency in μs and throughput in Mbits of DSLHash. The results are also compared with five lightweight hash functions selected from the NIST LWC finalists [54].

	Long		1563 bytes		64 bytes		16 bytes	
	Latency	Throughput	Latency	Throughput	Latency	Throughput	Latency	Throughput
Ascon1a_v1 [53]	29.64	844	14.85	842	0.99	515	0.55	233
Ascon1b [55]	31.79	787	15.92	785	0.94	543	0.52	248
Xoodoo_R2 [56]	16.40	1525	8.25	1515	0.50	1024	0.25	507
PHOTON-Beetle [57]	50.65	494	25.67	489	1.64	313	0.30	423
SPARKLE [58]	44.44	563	22.27	562	1.45	353	0.70	184
DSLHash0	48.71	513	24.40	512	1.63	313	0.90	142
DSLHash1	55.47	451	27.78	450	1.86	275	1.03	121
DSLHash2	61.56	406	30.84	405	2.07	248	1.14	112
DSLHash3	61.56	406	30.84	405	2.07	248	1.14	112

## Data Availability

The source code and supporting materials for the implementation and evaluation of DSLHash are available at [https://gitee.com/zhangjunhbxf/dslhash.git] (accessed on 12 June 2026). These resources are provided to facilitate independent verification and reproducibility of the reported results.
